# The evolution of folate supplementation – from one size for all to personalized, precision, poly-paths

**DOI:** 10.2478/jtim-2023-0087

**Published:** 2023-07-05

**Authors:** Qiangqiang He, Jianping Li

**Affiliations:** Graduate School at Shenzhen, Tsinghua University, Shenzhen 518055, Guangdong Province, China; Shenzhen Evergreen Medical Institute, Shenzhen 518057, Guangdong Province, China; Department of Cardiology, Peking University First Hospital, Beijing 100871, China

**Keywords:** folate, homocysteine, 5-methyltetrahydrofolate, vitamin D, precision nutrition

## Abstract

Folate is a crucial nutrient that supports physiological functions. Low folate levels is a risk factor for several diseases, including cardiovascular diseases and neural tube defects. The most used folate supplement is folic acid, a synthetic oxidative form, and folic acid grain fortification is a success story of public health. However, the metabolic conversion of folic acid to bioactive tetrahydrofolate requires several enzymes and cofactors. Therefore, these factors influence its bioavailability and efficacy. In contrast, 5-methyltetrahydrofolate is used directly and participates in one-carbon metabolism, and the use of 5-methyltetrahydrofolate as an alternative folate supplement has increased. The metabolism of 5-methyltetrahydrofolate is primarily dependent on the transmembrane transporter, reduced folate carrier (*RFC*), and the *RFC* gene *SLC19A1* variant is a functional polymorphism that affects folate status indexes. Recent studies demonstrated that the expression of *RFC* and cystathionine β-synthase, another enzyme required for homocysteine clearance, increases significantly by supplementation with calcitriol (vitamin D3), suggesting that calcitriol intake promotes the bioavailability of folate and has synergistic effects in homocysteine clearance. The advancements in biomedical and cohort studies and clinical trials have enhanced our understanding of the critical roles of folate and the regulation of one-carbon metabolism. We anticipate that the field of folate supplementation is poised to evolve from one size for all to personalized, precision, poly-paths (3Ps), which is a critical measure to meet individual needs, maximize health benefits, and minimize side effects.

## Introduction

Folate is the generic term for the water-soluble B-complex vitamin B9. The term folate stems from the Latin word *folium*, which means leaf; thus, folates are present in substantial amounts in green leafy vegetables.^[1]^ Many vegetables and legumes are rich sources of folate, with the concentrations being around 200 pg/ 100 g in leafy vegetables and up to 600 pg/100 g in some beans and chickpeas. In contrast, meat and meat products, except for the liver, contain negligible amounts of folate.^[2]^ The most oxidized and stable form is folic acid, a synthetic form of folate, which is composed of three covalently linked components: a pteridine ring, *p*-aminobenzoic acid, and a glutamate residue.^[3]^ Mammals can synthesize the pteridine ring, but cannot couple this ring to other compounds, and are thus dependent on folate uptake from exogenous sources.^[4]^

Adequate folate intake is essential, as folate is a crucial cofactor in one-carbon metabolism and plays a vital role in methylation reactions. Folate deficiency is associated with elevated plasma concentrations of homocysteine (Hcy), which is demonstrated to be a risk factor or marker of many diseases and disorders.

The purpose of this review is to summarize the advancements in folate supplementation and fortification over the past two decades and the latest viewpoints from experimental and clinical studies, which provide an evolutionary history of precision nutrition.

## Overview: The Functions and Requirements of Folate

Folate is a water-soluble B vitamin that plays a critical role in nucleic acid biosynthesis, DNA repair, and methylation.^[5]^ Adequate folate intake is vital for cell division and homeostasis because folate coenzymes play essential roles in nucleic acid synthesis, methionine regeneration, and the shuttling, oxidation, and reduction of one-carbon units required for normal metabolism and regulation.^[6]^ Low folate status is associated with elevated plasma Hcy, which are both risk factors for cardiovascular disease,^[7,8]^ stroke,^[9,10]^ megaloblastic anemia, neural tube defects (NTDs; congenital malformation of the fetus),^[11]^ depression,^[12]^ Alzheimer’s disease,^[13,14]^ and tumors.^[15]^

Food folates exist as pteroyl polyglutamates and must be hydrolyzed in the gut to monoglutamates before absorption.^[16]^ 5-Methyltetrahydrofolate (5-MTHF) is the predominant form of dietary folate and the only form of folate normally found in circulation. Thus, this is the form that is typically transported to the peripheral tissues for use in cellular metabolism. 5-MTHF accounts for approximately 98% of folate in human plasma.^[17]^

The daily Dietary Reference Intake of folate for adults is 400 μg of dietary folate equivalents.^[18]^ However, many people do not have sufficient natural folate in their diet, especially populations with high folate demands, such as pregnant and lactating women. Historically, folic acid deficiency has been a global problem, and even today, it remains a severe problem in countries where dietary supplementation is not mandatory. Maintaining adequate folic acid levels requires sufficient intake, efficient intestinal absorption, and a specific transport system to achieve optimal tissue distribution.^[19]^ Folic acid supplementation and fortification are the first choices to target folate deficiency as the most effective direct approach.

## Metabolic Pathway of Folate

One-carbon metabolism encompasses a complex metabolic network based on the biochemical reactions of folate compounds. Folic acid is a hub of one-carbon metabolism that activates and transfers methyl groups for biosynthesis, including purine and thymidine syntheses.^[20]^ However, folic acid has no coenzyme activity and must be reduced to the metabolically active form, 5-MTHF, through a series of steps. First, folic acid is reduced to dihydrofolate (DHF) and then tetrahydrofolate (THF) by dihydrofolate reductase (DHFR), which is converted to the bioactive form 5-MTHF by methylenetetrahydrofolate reductase (MTHFR).^[21]^

**Figure 1:**
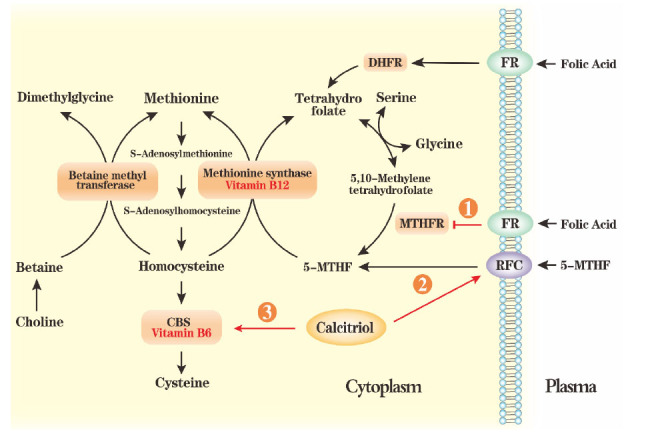
An overview of one-carbon metabolism and the folate cycle. One-carbon metabolism mainly involves the folate and homocysteine cycles. Plasma folic acid enters the tissue cells via FR, and then DHFR converts it into tetrahydrofolate. Next, tetrahydrofolate is transformed into 5,10-methylenetetrahydrofolate. Then, MTHFR converts 5,10-methylenetetrahydrofolate into 5-MTHF, providing a methyl group for converting homocysteine into methionine in a reaction catalyzed by MTR, which needs vitamin B12 as a cofactor. 5-MTHF enters into cells via RFC. Betaine, the end-product of oxidative metabolism of choline, is an alternative methyl donor for methylating homocysteine, which is catalyzed via BHMT. In the transfer-sulfuration pathway, CBS produces cysteine, which requires vitamin B6 as a coenzyme mediator. ①**: Folic acid as an inhibitor of MTHFR;** ②**: Calcitriol as an enhancer of RFC;** ③: Calcitriol as an activator of CBS. 5-MTHF: 5-methyl-tetrahydrofolate; BMT: betaine–homocysteine methyltransferase; CBS: cystathionine β-synthase; DHFR: dihydrofolate reductase; FR: folate receptor; MTHFR: methylenetetrahydrofolate reductase; MTR: methionine synthase; RFC: reduced folate carrier.

Hcy is a sulfur-containing, non-protein, vascular, toxic amino acid located at a branch point within the one-carbon metabolic pathway. Hcy is metabolized via two different pathways: (i) irreversibly degradation via the transsulfuration pathway to cysteine and (ii) remethylated back to methionine via the remethylation pathway (Figure 1). The latter process is disrupted by insufficient or metabolic abnormalities of folic acid levels and a deficiency in vitamin B12, which can eventually lead to hyperhomocysteinemia. As confirmed by epidemiological studies, high Hcy levels increase the risk of hypertension significantly and cardiovascular and cerebrovascular diseases.^[8,22]^

In the transfer-sulfuration pathway, cysteine is produced by the action of cystathionine β-synthase (CBS), a process that requires vitamin B6 as a coenzyme mediator.^[23,24]^ 5-MTHF serves as a methyl donor in the remethylation pathway. Under the action of methionine synthetase and with vitamin B12 as a coenzyme, Hcy is methylated to methionine^[25]^ and further transformed into *S*-adenosyl methionine (SAM). SAM is an essential active methyl donor and the second most common enzyme cofactor after adenosine triphosphate (ATP). SAM plays a significant role in epigenetics and biosynthetic processes, including phosphatidylcholine, creatine and polyamine syntheses, and sulfur metabolism.^[26,27]^ This cross-linking of folic acid and methionine cycles results in an inverse relationship between Hcy and folic acid levels. Thus, plasma Hcy is regarded as a functional indicator of folate status in the body.^[28]^

The Northern Manhattan Study affirmed that elevated total Hcy (tHcy) is a significant and robust predictor for ischemic stroke and vascular death.^[29]^ Clearance of tHcy conferred health benefits at all levels, but the lowest tHcy levels enjoyed the most significant risk reduction.

## Folic Acid Supplements

Folic acid (pteroylmonoglutamate) is absorbed, whereas food folates (polyglutamate derivatives) must be hydrolyzed to monoglutamates in the gut by a brush border hydrolase before absorption.

Folate supplements include their oxidized form, folic acid, and reduced form, mainly 5-MTHF or 5-MTHF calcium salt, which is commercially available as a crystalline form. Food folates exist primarily as 5-MTHF and formyltetrahydrofolate; however, native folates are generally polyglutamates, where each glutamate residue is linked to another by a γ-glutamyl bond. Polyglutamyl folates are hydrolyzed to folylmonoglutamates by pteroyl-γ-glutamyl hydrolase and then metabolized within enterocytes to 5-MTHF.^[30]^

Folic acid is used widely as a supplement and in food fortification because of its low price, high thermostability, and high bioavailability.^[31]^ Nonetheless, folic acid lacks coenzyme activity and must be reduced to the metabolically active form, tetrahydrofolate, within cells. 5-MTHF is the predominant form of dietary folate and the only species normally detected in the circulation and used for cellular metabolism.

5-MTHF and folic acid have similar physiological activity, bioavailability, and absorption at equimolar doses. Bioavailability studies have measured blood concentrations of folate and functional indicators of folate status and plasma Hcy and concluded that 5-MTHF is at least as effective as folic acid in improving the folate status.

Some countries have mandated the fortification of flour with folic acid or other vitamins and minerals (USA 1.4 mg/kg from 1998; Canada 1.5 mg/kg from 1998; Chile 2.2 mg/kg from 2000).[32] Currently, 92 countries have legislation to mandate fortification of at least one industrially milled cereal grain (Table 1); 91 of these countries requested wheat flour fortification, either alone or with other grains.[33] The benefits of folic acid supplementation are apparent: in the USA, Canada, and Chile, mandatory fortification of flour substantially improved folate and Hcy status and the NTDs’ rates fell by between 31% and 78%.[34] A recent study estimated that 65,380 congenital disabilities of the brain and spine were prevented in 2019 because the flour was fortified with folic acid.^[35]^ This causality was further demonstrated in an observational study that identified significant reduction in the risk of congenital heart disease and cleft lip and palate in children whose mothers received folic acid supplements or multivitamins.^[36]^

**Table 1:** Mandatory fortified cereal grain and the number of countries with corresponding legislation – July 2022^[30]^

Mandatory fortified cereal grain	Number
Wheat flour	67
Wheat flour, maize flour	17
Wheat flour, rice	5
Wheat flour, maize flour, rice	2
Rice	1

Over the last two decades, folic acid supplementation doses have been defined using general population characteristics. During this period, official agencies such as the World Health Organization (WHO) and the European Food Safety Agency (EFSA) specifically distinguished between the fortification requirements of different groups, including infants, children, adults, pregnant women, and others. Another consideration for folic acid dosage recommendation is the presence or lack of supplementation or food fortification.^[37]^ Alternatively, some studies have used Hcy as an evaluation index for supplemental folic acid dosage.^[28,38]^

As previously mentioned, vitamin B6 and B12 participate as coenzymes in the metabolism of folic acid and Hcy. For this reason, folic acid supplementation alone may not be able to maximize the reduction of Hcy levels. Related studies have explored the optimization of folic acid status indicators with folic acid supplementation, as well as vitamin B6 and B12. The results showed that at 0.8 mg/d folic acid, the Hcy levels were reduced by 23%. Additional supplementation with vitamin B12 was associated with a further reduction in Hcy by 7%.^[35]^ Another study reported that folic acid supplementation lowered fasting tHcy by 19.6% (*P* < 0.001), and that following folic acid supplementation, low-dose vitamin B6 lowered fasting plasma tHcy by 7.5%.^[39]^ A systematic meta-analysis of prospective studies to examine the relationship between dietary (from diet and supplements) intake of these B vitamins and the risk of stroke (with 12 prospective studies comprising 389,938 participants and 10,749 cases included in the final analysis) indicated that both vitamin B6 and folate intake were inversely correlated with the risk of stroke. Each 0.5 mg/d increase in vitamin B6 intake reduced the risk of stroke by 6%.^[40]^ This observation shows that when used in combination with vitamin B12, B6, and other B vitamins, folic acid can achieve a greater level of Hcy reduction than when used alone.

## Reduced Folate Supplement

Previous studies have indicated that single-nucleotide polymorphisms (SNPs) in genes encoding enzymes of one-carbon metabolism are crucial determinants of Hcy concentration.^[41]^ A recent study confirmed that one-carbon metabolism gene variants affect the response to folate treatment.^[42]^

In 1995, Frosst *et al*.^[43]^ identified a common mutation in *MTHFR* : a C to T substitution at bp 677. This mutation alters a highly conserved amino acid, and in both heterozygous and homozygous individuals, it causes a reduction in enzyme activity and increased thermolability. Individuals homozygous for the mutation (*MTHFR* 677TT) have significantly elevated Hcy^[43,44]^ and altered folate distribution within red blood cells.^[44]^ An increased circulating concentration of Hcy is associated with a higher risk of stroke.^[9]^ Further investigations have shown that the mutation frequency of C677T is higher among the Chinese population when compared to the European and American populations.^[45]^ Research has suggested that TT individuals require higher folate intake than those with the CT or CC genotype to counterbalance the effect of the *MTHFR* 677 mutation on tHcy levels and achieve similar tHcy concentrations.^[45,46]^ The consensus is that the precise folic acid supplementation dose should be based on individual genotypes. Although accurate precision supplementation has not yet been realized, progress has been made through the subdivision of populations by age and genotype combined with other factors.

The average dose of folic acid expected from food fortification in the USA and Canada is approximately 100 μg/d, which is mainly converted to 5-MTHF.^[3]^ However, an important issue has emerged from research on folic acid supplementation: above 200 μg/d, small amounts of folic acid appear unchanged in the circulation.^[47]^ Thus, people taking supplements or consuming fortified foods may face a buildup of unmetabolized folic acid (UMFA).^[48]^ Before the body uses folic acid, it must first be reduced to DHF and subsequently to THF. Production of DHF and THF is catalyzed by DHFR in the liver.^[49]^ DHFR activity in the human liver has been shown to be low and highly variable.^[50]^ This observation points to a potentially limited ability to activate the synthetic form of folate and raises concerns about the clinical trials using high levels of folic acid (ranging from 0.5 to 15.0 mg/d).^[51]^ The extremely low rate of conversion of folic acid suggests that the potential benefits of its use in high doses are outweighed by saturation of DHFR, especially in individuals with lower-than-average DHFR enzyme activity.^[50]^ DHFR is saturated when folic acid intake exceeds a physiological threshold and conversion to the reduced form is impaired, resulting in the appearance of UMFA in circulation.^[25]^ The most publicized safety risk concerns the correcting and masking neurologic sequelae of cobalamin deficiency in patients exposed to UMFA, as UMFA may mask the underlying disease.^[52, 53, 54]^

Additionally, the presence of UMFA may cause accumulation of DHF. *In vitro*, DHF can inhibit the activities of MTHFR and thymidylate synthase, two enzymes required for one-carbon metabolism, theoretically leading to paradox functional folate deficiency.^[15]^ Given these risks, there has been growing interest in determining the amount of dietary folic acid required for UMFA to accumulate within the body. In one study, participants on a 5-day regimen of fortified, ready-to-eat-cereal and bread added to their regular diet had a threshold intake of 266 μg folic acid per meal for UMFA to appear in serum.^[47]^ Another study found that 1 mg of folic acid consumed in 10 equal doses of 100 μg also caused folic acid to appear in serum.^[55]^ Thus, UMFA was detected in people consuming either folic acid-fortified foods or supplements.^[48,56]^

Given the potential downsides of folic acid supplementation, there is a clear need for an alternative solution. Rather than using synthetic folate, that is, folic acid, a more direct option is to supplement with an already bioactive reduced form, such as 5-MTHF, which is the most available folate form in human plasma^[57]^ and constitutes 95%–98% of folate in serum.^[17,58]^

5-MTHF has many advantages over folic acid as a supplement. 5-MTHF participates directly in one-carbon metabolism without activation. Thus, 5-MTHF supplements should bypass the limitation of low DHFR activity and the decreased efficiency caused by several common polymorphisms of MTHFR and other enzymes. Unlike folic acid, data suggest that 5-MTHF will not mask vitamin B12 deficiency. 5-MTHF is also more effective than folic acid supplementation in improving folate status.^[59]^ Studies on the safety, tolerability, and retention rates of 5-MTHF/5-MTHF-Ca have suggested that 5-MTHF-Ca is a safe alternative to folic acid as a source of folate and may be particularly advantageous for individuals with MTHFR defects, who could have difficulty processing folic acid from supplements or fortified foods.^[60,61]^ Importantly, a study demonstrated that after administering 5-MTHF, UMFA was not detected in the plasma of women who were of MTHFR 677CT genotype.^[62]^ Products supplementing the active form of folic acid are also becoming increasingly abundant.

## Folate Transporters

As a coenzyme, folate is distributed primarily in the cytosol and mitochondria of liver cells, where one-carbon metabolism occurs.^[21]^ This requires folate molecules to cross membranes into these compartments. Because of the hydrophilic nature of the charged folate molecule, there is minimal passive diffusion across cell membranes. Instead, specific transporters are required to facilitate intestinal folate absorption and the transport of folates into systemic tissues.^[19]^

Three folate transporters account for folate influx activities observed in mammalian cells.

(1) Folate receptors (FRs) are membrane proteins with an endocytic transport mechanism that have a high affinity for folic acid (approximately 1 nmol/L) and a lower affinity for reduced folates.^[63]^ The genes encoding FR-α, -β, and -γ are located on chromosome 11 (q11.3–q13.5).^[64]^ FR-α is expressed in epithelial tissues. FR-β is expressed in cells of hematopoietic origin.^[65]^ FR-γ expression has been detected by polymerase chain reaction (PCR) analysis in normal and malignant hematopoietic cells present in the spleen, bone marrow and thymus, ovarian, cervical, and uterine carcinoma.^[66]^ The affinity of FRs for reduced folate cofactors and methotrexate (MTX) is approximately threefold and 100-fold lower, respectively, when compared to the affinity of FRs for folic acid.

(2) Proton-coupled folate transporter (PCFT) is a member of the superfamily of facilitative carriers (SLC46A1). From a physiological perspective, PCFT is required for intestinal folate absorption and transport of folates across the choroid plexus–cerebrospinal fluid barrier. PCFT has a very high affinity for folic acid at pH 5.5 and a low folic acid transport activity at pH 7.4.^[67]^

(3) Reduced folate carrier (RFC; SLC19A1) is an anion exchanger with a high affinity for reduced folates (*K*^m^ of 1–5 μmol/L) and a low affinity for folic acid (*K*^m^ of 200–400 μmol/L), with optimum activity at physiological pH.^[63]^

The human *RFC* gene is located on chromosome 21q22.3. Five exons encode RFC, which consists of 591 amino acids.^[68]^ The gene for this transporter is ubiquitously expressed in human tissues.^[69]^ RFC is a bidirectional folate transporter, functions as an anion exchanger, and is a major uptake route for transporting folates into mammalian cells at ambient neutral pH.^[70]^ RFC is a member of the solute carrier family (SLC) of facilitative carriers, which currently comprises 300 genes that have been classified into 43 subfamilies.^[1]^ The SLC family belongs to an even larger superfamily known as the major facilitator superfamily of transporters.^[71]^

5-MTHF, like all folates, is hydrophilic and incapable of permeating plasma membranes by diffusion alone. RFC is a high-capacity, bidirectional transporter of 5-MTHF, which mediates the transport of 5-MTHF from blood into the cells of peripheral tissues.^[72,73]^

## Regulation of Folate Transporters

Although 5-MTHF has apparent advantages over folic acid as a supplement, 5-MTHF is not a perfect solution. The *RFC* gene *SLC19A1* variant has been reported to have functional SNPs; the most extensively studied variant is G80A. This base change results in the exchange of arginine with histidine at residue 27 in the amino acid sequence. A study has shown that the *RFC* G80A polymorphism can impact folate status.^[73]^ In this study, women with the 80GA and 80AA genotypes had higher red blood cell folate concentrations when compared to women with the 80GG genotype,^[73]^ which is consistent with another study demonstrating that after stratification by the C677T genotype, individuals who were 80AA/677CT had higher plasma folate levels than those who were 80GG/677CT (*P* = 0.02).^[74]^ The *RFC* G80A polymorphism is ubiquitous among several ethnic populations. The G and A alleles at base 80 are distributed almost equally among Ashkenazi Jews, Hispanics, and Caucasians, with the A frequency ranging from 47.2% to 56.4%.^[75]^

Unfortunately, there is a paucity of data to illustrate the exact relationship between the G80A polymorphism of *RFC* and the folate status after 5-MTHF supplementation. We hypothesize that as variable activities of DHFR and polymorphisms of MTHFR and other enzymes are bypassed by supplementation with reduced folate, RFC, as an essential functional carrier of reduced folate, may become an important factor in defining dosage selection with 5-MTHF supplementation.

Indeed, the expression and regulation of RFC may interact with folate levels. One study showed that folate deficiency increased mRNA synthesis for folate transporters, including RFC and PCFT. This seems to be the underlying transcriptional regulatory mechanism controlling the expression of folate transporters,^[76]^ which may be a maintenance mechanism for folate metabolism homeostasis. Another study demonstrated that activation of vitamin D receptor (VDR) through calcitriol (1,25-dihydroxyvitamin D3) exposure upregulates *RFC* mRNA and protein expression and function in immortalized cultures of human cerebral microvascular endothelial cells (hCMEC/D3). Moreover, in isolated mouse brain capillaries, the role of VDR in the direct regulation of RFC was further confirmed. Subsequently, an *in vivo* study using Folr1-knockout (Folr1 KO) mice showed that a loss of FR-α led to a substantial decrease of folate delivery to the brain, and that pretreatment of Folr1 KO mice with the VDR-activating ligand, calcitriol, resulted in an over sixfold increase in the concentration of [^13^C5]-5-formyltetrahydrofolate ([^13^C5]-5-formyl THF) in brain tissues, with levels comparable to wild-type animals.^[77,78]^

The transsulfuration pathway is necessary for Hcy clearance and culminates in cysteine synthesis. The first step of this metabolic pathway is the condensation of Hcy and serine to form cystathionine, which is catalyzed by the vitamin B6-dependent enzyme CBS.^[79]^ In one study, a microarray experiment on MC3T3-E1 murine preosteoblasts treated with calcitriol revealed that the transcription and expression and the enzymatic function of CBS were rapidly and strongly induced by calcitriol.^[80]^

Calcitriol acts as a ligand for the nuclear transcription factor, VDR, regulating gene transcription and cell function. Calcitriol was found to regulate the transcription of about 3% of mouse and human genomes. Calcitriol metabolites may also function as hormones or cytokines for many cellular functions like cell cycle control and differentiation.^[81]^

These results suggest that calcitriol supplementation may enhance the bioavailability and bioefficacy of folate by improving its transport and activating one of the metabolic pathways for Hcy clearance. Therefore, folate combined with vitamin D intervention may be a therapeutic strategy to increase the absorption and distribution of folate in tissues of individuals with poor RFC activity or folate metabolism defects and promote the reduction of Hcy. This novel intervention aims to optimize folate supplementation by increasing its absorption and capacity in the body.

## Folate Supplement in Special Gro

***Folate supplementation for MTX-treated patients*** To establish individualized medication for patients receiving specific drug treatments, some issues need to be illustrated based on research data in this field.

MTX is a folate antagonist and a well-established therapy for autoimmune and cancer patients.^[82,83]^ RFC is the major route of folate and MTX uptake from extracellular fluids. A study showed that *RFC* mRNA expression levels are more important as a factor linked to MTX efficacy, and individual differences in *RFC* expression levels among subjects were observed. Therefore, individual differences in MTX treatment efficacy may be related to individual differences in *RFC* mRNA expression levels. Moreover, responsiveness to MTX may be predictable based on these individual differences in *RFC* mRNA expression.^[84]^ Additionally, the most studied SNP of RFC, G80A, was associated with altered MTX concentrations in leukemia patients treated with MTX,^[85]^ suggesting that the RFC 80A allele may potentiate the effect of low-dose MTX in patients with rheumatoid arthritis.^[86]^

In high doses, as used in cancer chemotherapy, MTX depletes the intracellular folate stores and thus disrupts nucleotide synthesis. MTX treatment is associated with significant side effects and toxicity in some patients. Folate supplementation is often used to ameliorate MTX-associated side effects and toxicities. According to studies reviewed, the use of folate supplements in patients treated with MTX reduces the incidence of hepatotoxicity and gastrointestinal intolerance without impairing the efficacy of MTX.

Furthermore, studies reported that MTX treatment may lead to increased plasma Hcy concentrations, and concomitant folate supplementation decreases plasma Hcy levels.^[87]^ Moreover, a study showed that low-dose MTX of 7.5–10.0 mg per week induces a significant rise in plasma Hcy during the first 4 weeks of treatment in arthritis patients without folic acid deficiency.^[88]^ However, relatively few studies have addressed folate supplementation with the use of MTX for treating psoriasis. Thus, folate supplementation has been recommended for every patient who receives MTX.^[89]^

In summary, folate reduces the adverse effects and toxicities without affecting the efficacy in studies of rheumatoid arthritis. The use of folate may increase the likelihood of efficacious long-term, tolerable, and toxicity-free therapy for patients receiving MTX.

### Folate supplementation for epileptic patients

The interaction between cytochrome P450 enzyme-inducing antiepileptic drugs (P450-inducing antiepileptic drugs [AEDs]) and folate metabolism may be clinically relevant and balanced by supplementation with small doses of folic acid. The results of a survey support the recommendation that women of childbearing age treated with AEDs receive folic acid supplementation, particularly those taking P450-inducing AEDs.^[90]^

Patients with epilepsy exhibit elevated plasma tHcy levels more frequently than the general population, which is caused by chronic treatment with older AEDs and polymorphisms in the *MTHFR* gene. Epileptic patients exhibit, in a percentage range of 20%–40%, supraphysiological plasma levels of tHcy; however, this tHcy level is only observed in approximately 5% of the general population.^[91]^ In another study, epileptic children with hyper-tHcy had significantly lower folate and cobalamin concentrations.^[92]^

Among the various variables analyzed, hyper-tHcy has been indicated as a risk factor for the progression of atherosclerosis in epileptic patients.^[93, 94, 95, 96, 97, 98]^ Noteworthy, literature data suggest that patients with epilepsy also exhibit an increased risk for stroke.^[99]^ In this sense, the definition of a standardized clinical assessment is essential for developing new therapeutic strategies, including folate and other B vitamin supplements, and reducing morbidity and mortality in epilepsy patients under long-term therapy.

Results have shown that the condition of hyper-tHcy can be easily corrected by the intake of folate and other B vitamins. This raises the question of how folic acid and/or B vitamins are provided to patients with epilepsy. Belcastro *et al*.^[100]^ summarized previous studies and claimed that supplementation of 0.4 mg/ d folic acid plus a low B vitamin dose (*i.e*., B6, B12) is an option to treat or avoid consequences of folate deficiency in patients on chronic treatment with older AEDs.

The above two cases reveal that people treated with drugs for nervous system disorders need carefully regulated and/or measured folate supplements to ensure benefits from them, when compared to the folate supplements used by the general population. Although the functional indicator of folate status, Hcy, is influenced by various factors such as genetic or environmental, as an independent risk of many disorders, monitoring Hcy levels deserves more attention by clinicians. Diversified and personalized solutions for this issue are promising, and significant progress and advances in this research field continue to be made.

## Conclusion

Over the past 20 years, universal folic acid grain fortification in many countries, including the USA, has been a success story in medicine and public health. Rapid advances in biomedical sciences (*e.g*., human genetics, epigenetics, proteomics, metabolomics) and high-throughput biotechnology, along with large-scale cohort studies and clinical trials, have greatly enhanced our understanding of the critical role and regulation of folate in the context of one-carbon metabolism. We anticipate that the field of folate supplementation is poised to evolve from one size for all to personalized, precision, poly-paths (3Ps). This evolution is critically needed to meet individual needs, maximize health benefits, and minimize adverse effects. Well-designed clinical trials and prospective cohort studies will be required to deepen our knowledge and pave the road toward 3Ps. Findings from this line of research will be transformative and will shape the future of 3P folate nutrition.
